# Anti-TNF-Mediated Modulation of Prohepcidin Improves Iron Availability in Inflammatory Bowel Disease, in an IL-6-Mediated Fashion

**DOI:** 10.1155/2017/6843976

**Published:** 2017-01-16

**Authors:** Flaminia Cavallaro, Lorena Duca, Laura Francesca Pisani, Roberta Rigolini, Luisa Spina, Gian Eugenio Tontini, Nadia Munizio, Elena Costa, Maria Domenica Cappellini, Maurizio Vecchi, Luca Pastorelli

**Affiliations:** ^1^Gastroenterology and Gastrointestinal Endoscopy Unit, IRCCS Policlinico San Donato, San Donato Milanese, Italy; ^2^Department of Clinical Sciences and Community, University of Milan, Milan, Italy; ^3^Department of Internal Medicine, IRCCS Ca' Granda Ospedale Maggiore Policlinico, Milan, Italy; ^4^Laboratory Medicine Service, IRCCS Policlinico San Donato, San Donato Milanese, Italy; ^5^Department of Biomedical Sciences for Health, University of Milan, Milan, Italy

## Abstract

*Background.* Anaemia is common in inflammatory bowel disease (IBD), frequently resulting from a combination of iron deficiency and of anaemia of chronic disease (ACD). ACD is characterized by macrophage iron retention induced by proinflammatory cytokines. Hepcidin is the master inducer of iron accumulation during ACD, and its production is mainly regulated by IL-6 and the novel erythroid hormone erythroferrone (ERFE). This study evaluates whether anti-TNF monoclonal antibodies therapy modurates hepcidin production and the levels of its main regulators, leading to a restoration of iron homeostasis.* Methods.* Sera were collected from 21 IBD patients, before each anti-TNF administration, for the first 6 weeks of therapy. Prohepcidin, erythropoietin, erythroferrone, C reactive protein, interleukin-6, iron markers, and haemoglobin levels were measured and clinical activity indexes were evaluated.* Results.* Serum prohepcidin, IL-6, CRP, and ferritin were significantly reduced after 6-week treatment; an increase in serum iron and total transferrin was observed. No changes in the EPO-ERFE axis were found. Remarkably, haemoglobin was significantly increased.* Conclusions.* Anti-TNF therapy improves iron metabolism and, subsequently, anaemia in IBD. This effect appears to be related to the modulation of the cytokine network and specifically IL-6 leading to a relevant decrease of hepcidin, a master regulator of ACD.

## 1. Introduction

Anaemia is a common systemic manifestation of inflammatory bowel disease (IBD), occurring in 6% to 74% of patients [1]. Anaemia in IBD is a prototypic combination of iron deficiency and anaemia of chronic disease, but vitamin deficiencies and myelosuppressive drugs, such as thiopurines and/or methotrexate, may also play a role [2].

Iron deficiency in IBD maybe a consequence of chronic/recurrent bleeding from ulcerated intestinal mucosa; in Crohn's disease (CD), it may also be associated with iron malabsorption, due to an impaired absorptive function in the inflamed small bowel [3, 4].

Laboratory tests in iron deficiency anaemia usually depict a classical panel characterized by low serum levels of iron and ferritin, reduced transferrin saturation, and increased transferrin concentration.

On the other hand, anaemia of chronic disease (ACD) is characterized by normal or increased ferritin levels, as a result of increased storage and retention of iron within the reticuloendothelial system; in fact, during chronic inflammatory diseases proinflammatory cytokines lead to the activation of macrophages which augment their erythrophagocytic activity and express increased levels of divalent metal transporter-1 (DMT-1), a transmembrane protein functioning as a major iron uptaker. Conversely, the macrophage expression of ferroportin-1, the only known cellular iron exporter, is reduced, blocking the release of iron from these cells and ultimately leading to intracellular iron accumulation [5].

Recent data suggest that hepcidin, an acute phase protein produced by the liver, is a major regulator of iron metabolism. In fact, hepcidin inhibits the function of ferroportin-1, expressed by macrophages and enterocytes; thus, high levels of hepcidin favour iron storage in the reticuloendothelial system and reduce iron absorption from the gut, promoting the development of ACD [5].

Hepcidin expression is mainly induced by the proinflammatory cytokine interleukin-5 (IL-6) and by the bacterial lipopolysaccharide. More recently, a peptide previously known as Fam 132b has been recognized to negatively regulate hepcidin synthesis and named erythroferrone (ERFE) [6]. In a murine model, it has been shown that, after haemorrhage, ERFE-mediated suppression of hepcidin allows increased iron absorption and mobilization from stores. In fact, ERFE mediates hepcidin downregulation during erythropoiesis and itis produced by erythroblasts in the bone marrow and in the spleen in response to erythropoietin (EPO) [6].

Indeed, hepcidin regulation is finely tuned by opposing stimuli: on one hand by proinflammatory molecules which enhance its production and lead to ACD during inflammatory/infectious conditions on the other hand, by the EPO-ERFE axis, which, according to recent data, appears to keep hepcidin suppressed in order to recover from a hypoxic state and to restore erythropoiesis.

Among the many pr-inflammatory cytokines, tumour necrosis factor- (TNF-) *α* plays a major pathogenic role in immune-mediated disorders such as inflammatory bowel diseases. Indeed, anti-TNF monoclonal antibodies (namely, Infliximab and Adalimumab) are effective therapeutic options in inducing remission in moderate to severe IBD, throughout the downregulation of several proinflammatory mediators.

Aim of the study was thus to evaluate whether anti-TNF agents exert any effect on hepcidin production and on its regulators, leading to a restoration of normal iron homeostasis in IBD patients.

## 2. Patients and Methods

### 2.1. Patients

IBD patients (16 CD and 5 UC) scheduled to undergo anti-TNF therapy with Infliximab (9 CD and 5 UC) or Adalimumab (7 CD) at the Gastroenterology and Endoscopy Unit of IRCCS Policlinico San Donato were enrolled, after having read and signed a specific informed consent. All diagnoses had been confirmed by clinical, endoscopic, and histologic criteria [7, 8]. In the whole population, the mean age of the patients was 40 years (range 18–62) and there were 7 men and 14 women. None of the patients was treated with blood transfusions, iron, or vitamin supplementations at the moment of enrollment and in the following weeks. The indications to anti-TNF therapy were corticosteroid-dependent/resistant active disease (19 patients) or perianal disease (2 patients). Disease activity was assessed using the Harvey-Bradshaw Index (HBI) for CD patients [9] and the Mayo scores for UC [10].

The demographic and clinical characteristics of the patients are reported in Table 1.

All patients underwent blood sampling 1 h prior to each drug administration, with the following schedules: *T*_0_, week 2 and week 6 for Infliximab infusions (5 mg/kg/infusion); *T*_0_, week 2, week 4, and week 6 for Adalimumab injections (160-80-40-40 mg/injection). The two different schedules of sampling were related to the different induction therapy regimens characteristic of the two drugs. A reduction of at least two points in the original HBI or in the partial Mayo scores (*i.e.,* Mayo score without endoscopic evaluation), by the end of the induction regimen, was considered indicative of clinical response to therapy.

### 2.2. Serum Collection

Whole blood samples were used to measure whole blood cell count. Peripheral blood was also collected by venipuncture of an anticubital vein without any blood stasis using sterilized needles in BD vacutainer® SST™ II Advance for serum collection. A 3 ml blood tube was used for serum separation after centrifugation at 2500 ×g for 20 min at room temperature. Serum was stored at −80°C until the time of assays.

### 2.3. Prohepcidin and Iron Status Markers' Measurement

Prohepcidin, a dosable hepcidin precursor, was measured by means of a commercially available ELISA kit (DRG, Marburg-Germany).

Serum ferritin was determined by a solid-phase two-site chemiluminescent-immunometric assay. Transferrin and C reactive protein (CRP) levels were measured by an immunoturbidimetric assay and iron by a colorimetric assay (Roche Diagnostic, Mannheim, Germany).

### 2.4. Erythroferrone (ERFE), Erythropoietin (EPO), and Interleukin- (IL-) 6 Concentration

Erythroferrone (ERFE) and interleukin- (IL-) 6 levels were measured by means of commercial sandwich ELISA kits according to the manufacturers' instructions (Cusabio Biotech, China; Abnova, UK resp.).

Erythropoietin was determined by IMMULITE 2000 EPO a chemiluminescent-immunometric assay (Siemens Healthcare, Italy).

### 2.5. Statistical Analysis

Statistical analysis was performed by means of paired Student's *t*-test and Pearson's correlation test.

### 2.6. Ethical Requirements

The Internal Review Board of the local Ethical Committee approved the study (Ethical Committee Protocol number 2025, ASL Milano-2, approved on June 14, 2007), in accordance with the 1964 Declaration of Helsinki.

## 3. Results

### 3.1. Clinical Response to Anti-TNF Therapy

**Figure 1:**
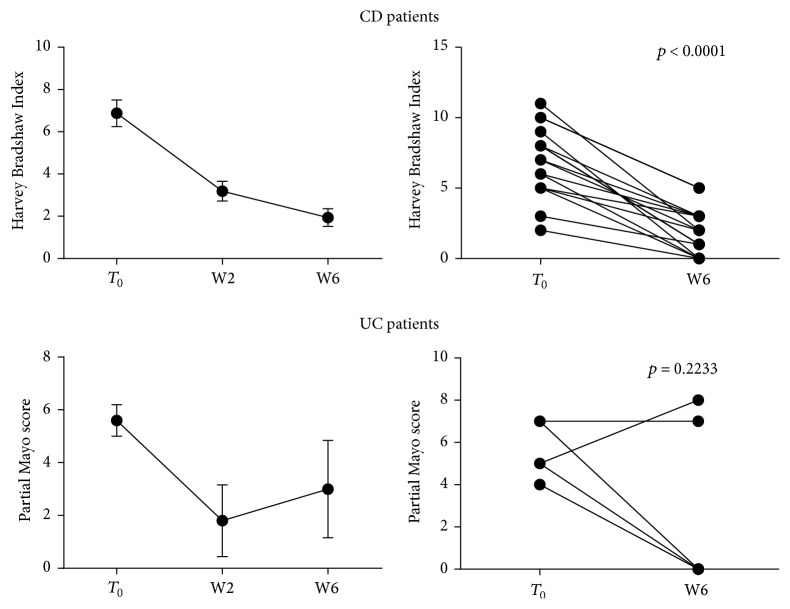
Clinical activity indexes throughout anti-TNF therapy. Harvey-Bradshaw Index and partial Mayo score were calculated before each drug administration to CD (*N* = 16) and UC (*N* = 5) patients, respectively, undergoing anti-TNF therapy. Statistical analysis was performed using Student's *t*-test for paired data. Data are presented as single data point or as mean ± SEM.

In order to evaluate the response to anti-TNF therapy we calculated HBI and partial Mayo scores for CD and UC patients, respectively, at baseline, that is, before the first Infliximab or Adalimumab administration (*T*_0_), and at the end of the observation period, that is, before the week 6 of drug administration schedule (W6). All patients affected by CD showed a clinical response to anti-TNF infusion, that is, a reduction of at least two points in the original HBI (mean HBI 6.87 ± 0.63* versus*  1.93 ± 0.41 at *T*_0_ and W6, resp.; *p* < 0.0001) (Figure 1 and Table 2).

Three out of five UC patients treated with Infliximab had a complete clinical response (final partial Mayo = 0), whereas two did not achieve any benefit from the aforementioned therapy (Figure 1, Table 2).

### 3.2. Pharmacologic Block of TNF, Dampening Systemic Inflammation, Reduces Prohepcidin Serum Levels

After establishing the overall good response to therapy, we wanted to evaluate whether or not it corresponded to any change in the expression of a master regulator of iron metabolism, that is, hepcidin. Therefore, we measured serum concentration of prohepcidin, a circulating precursor of hepcidin, which is easily detected in the serum and correlates with actual hepcidin levels [11, 12]. Blood samples were collected before each drug administration, throughout the therapy schedule, from *T*_0_ to W6.

**Figure 2:**
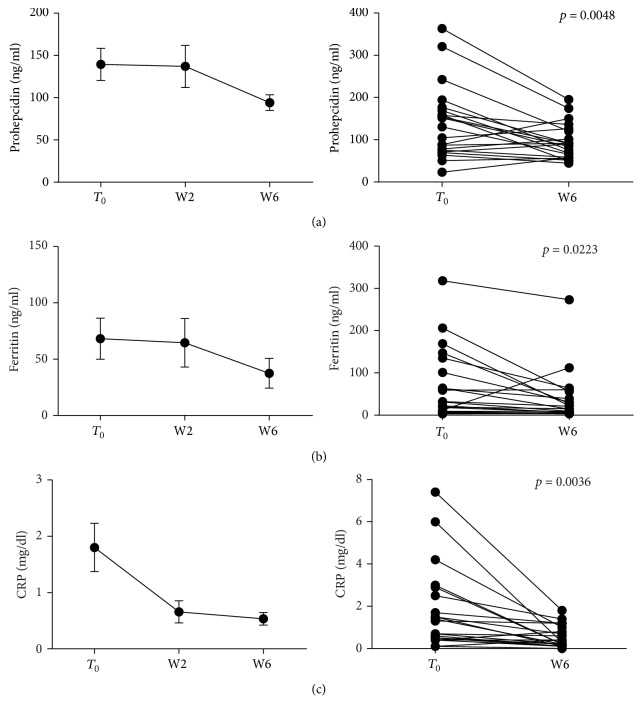
Prohepcidin and acute phase protein levels during anti-TNF therapy. Serum samples were collected before each anti-TNF administration and prohepcidin, ferritin, and CRP circulating levels measured. Statistical analysis was performed using Student's *t*-test for paired data. Data are presented as single data point or as mean ± SEM.

When all patients (both CD and UC) treated with anti-TNF were analyzed, serum levels of prohepcidin at W6 were significantly decreased, compared with levels measured before *T*_0_ administration (139.42 ± 18.96* versus*  94.14 ± 9.19 ng/ml, *p* = 0.0048); consistently, circulating levels of other acute phase proteins, such as ferritin and CRP, were also reduced (68.19 ± 18.23* versus*  37.48 ± 13.22 ng/ml, *p* = 0.0223 and 1.80 ± 0.42* versus*  0.53 ± 0.11 mg/dl, *p* = 0.0036, resp.). All these data are reported in Figure 2.

**Figure 3:**
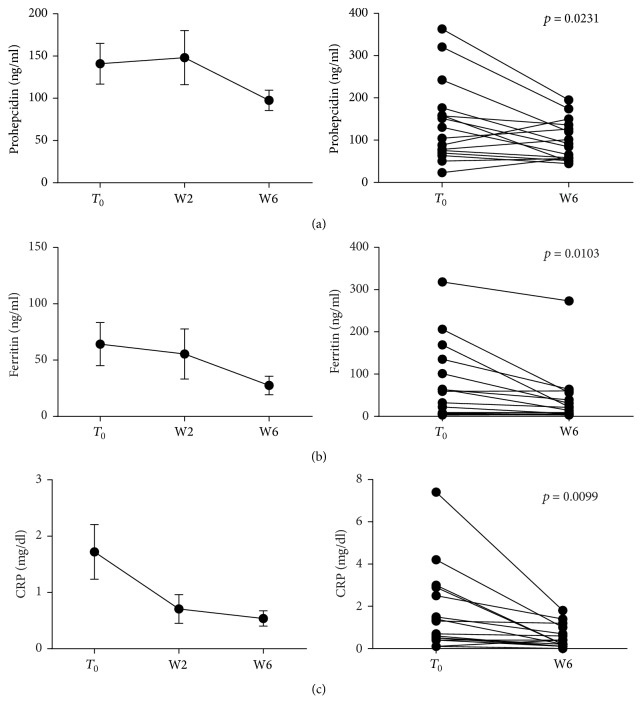
Prohepcidin and acute phase protein levels during anti-TNF therapy in CD patients. Blood samples were collected before each anti-TNF administration and parameters measured. Statistical analysis was performed using Student's *t*-test for paired data. Data are presented as single data point or as mean ± SEM.

**Figure 4:**
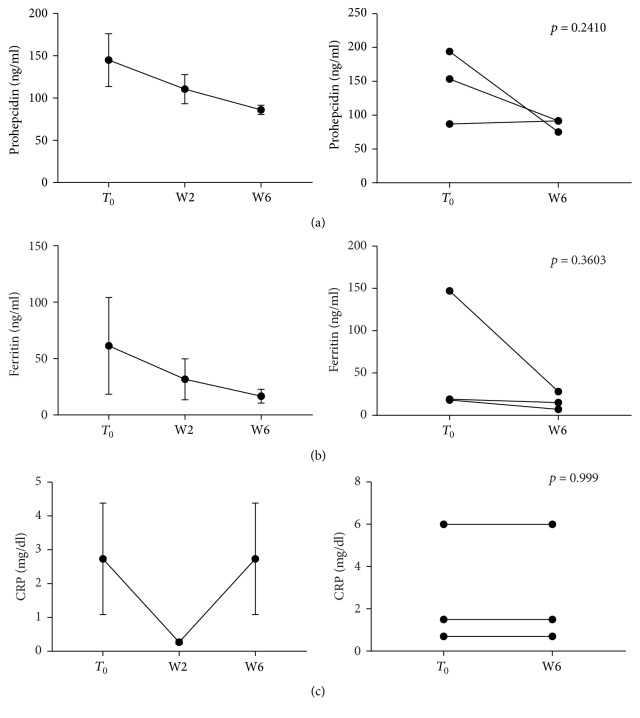
Prohepcidin and acute phase protein levels during anti-TNF therapy in UC patients. Blood samples were collected before each anti-TNF administration and parameters measured. Statistical analysis was performed using Student's *t*-test for paired data. Data are presented as single data point or as mean ± SEM.

When stratifying patients according to disease, significant reductions of prohepcidin, CRP and ferritin were detected in CD patients (*p* = 0.0231, *p* = 0.0099, and *p* = 0.0103, resp.) (Figure 3), whereas UC patients only showed a trend towards a decrease of prohepcidin and CRP (Figure 4).

When different drugs were considered, prohepcidin and CRP were significantly reduced in IFX treated patients (*p* = 0.0318 and *p* = 0.0112, resp.), while ferritin showed a nonstatistically significant decrease (*p* = 0.0651) (Supplementary Figure 1 in Supplementary Material available online at https://doi.org/10.1155/2017/6843976); on the other hand, in the ADA treatment group whereas we demonstrated reductions of all the aforementioned parameters, none of these reached the statistical significance (Supplementary Figure 2).

### 3.3. Anti-TNF Administration Restores Iron Availability and Usage in IBD Patients

**Figure 5:**
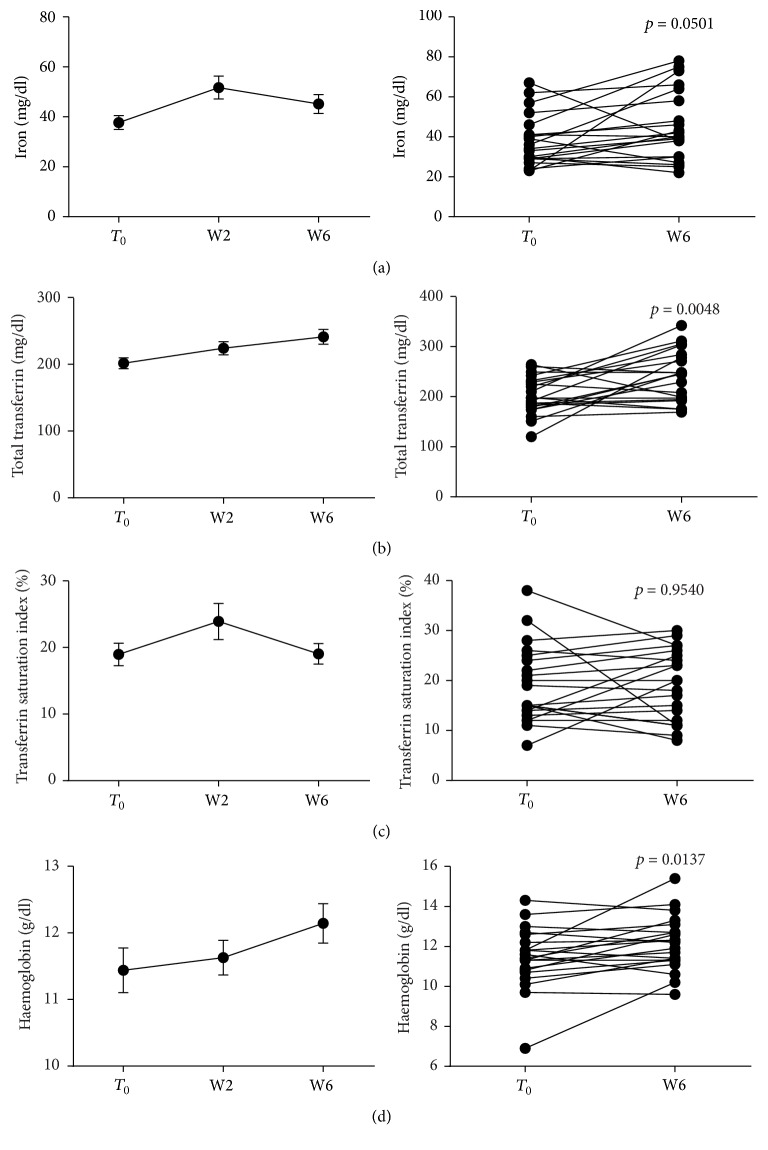
Modulation of iron markers and haemoglobin following anti-TNF therapy. Blood samples were collected before each anti-TNF administration (*N* = 21/time point) and circulating iron, transferrin, and haemoglobin levels measured. Transferrin saturation was calculated, as well. Statistical analysis was performed using Student's *t*-test for paired data. Data are presented as single data point or as mean ± SEM.

As anti-TNF therapy reduced circulating prohepcidin and ferritin, we detected a substantial improvement of iron homeostasis; in fact, following Infliximab or Adalimumab administration, circulating iron increased significantly from *T*_0_ to W6 (37.71 ± 2.77* versus*  45.14 ± 3.78 *μ*g/dl, *p* = 0.0501) (Figure 5). We also witnessed a significant increase of total transferrin (201.5 ± 8.12* versus*  241.2 ± 10.99 mg/dl, *p* = 0.0048), which is consistent with the reduction of systemic inflammatory activation (Figure 5). No significant changes in transferrin saturation were observed (Figure 5). Remarkably, haemoglobin levels significantly increased when comparing baseline values at *T*_0_ with those observed at W6 (11.44 ± 0.33* versus*  12.14 ± 0.29 g/dl, *p* = 0.0137), as reported in Figure 5, suggesting an increase in the availability and usage capacity of circulating iron. When we analyzed data clustering patients for disease, transferrin and haemoglobin levels were significantly increased in CD patients (*p* = 0.0033 and *p* = 0.0115, resp.) (Figure 3); instead, in UC patients, no significant changes in circulating iron, transferrin, and haemoglobin were detected (Figure 4). When stratifying patients for drug used, all those parameters were significantly increased in IFX treated patients (*p* = 0.0239, *p* = 0.0070, and *p* = 0.0048 for iron, transferrin, and haemoglobin, resp.) (Supplementary Figure 1), but not in ADA group (Supplementary Figure 2).

### 3.4. Anti-TNF Blockade Reduces IL-6 Levels but Does Not Modulate EPO-ERFE Axis

**Figure 6:**
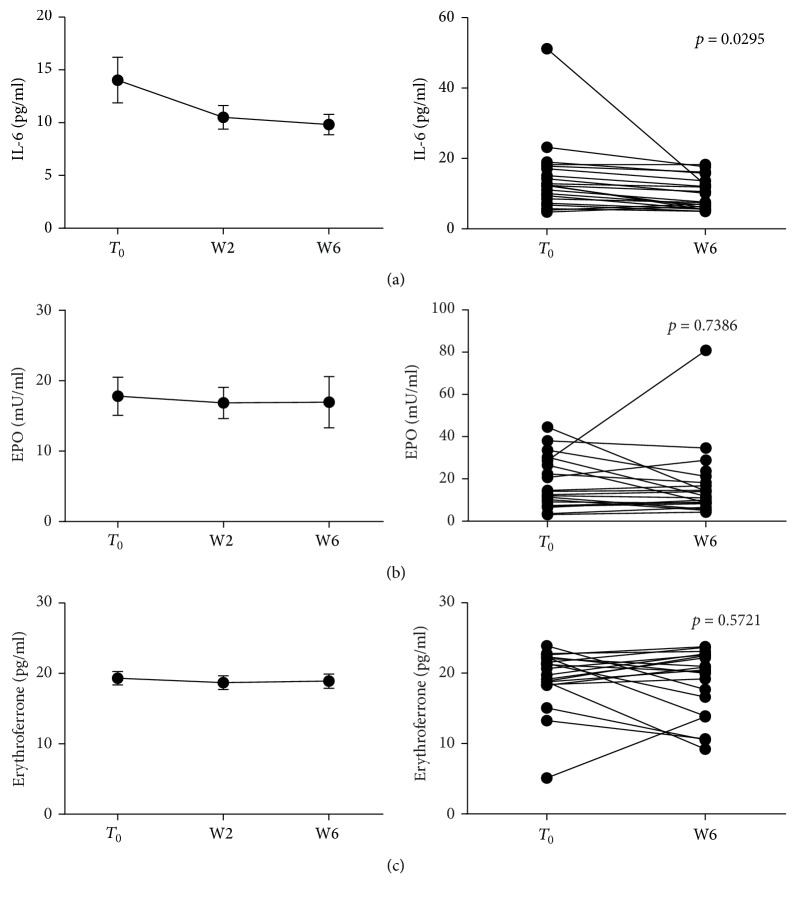
IL-6, EPO, and ERFE levels before and after 6 weeks of anti-TNF therapy. Sera were collected before each anti-TNF administration (*N* = 21/time point) and IL-6, EPO, and ERFE levels measured. Statistical analysis was performed using Student's *t*-test for paired data. Data are presented as single data point or as mean ± SEM.

We investigated the role of anti-TNF drug administration in modulating the main regulators of hepcidin production; thus, we measured IL-6, EPO, and ERFE circulating levels before and following the first 6 weeks of Infliximab/Adalimumab therapy. IL-6 levels were significantly reduced from *T*_0_ to W6 (14.03 ± 2.15* versus*  9.82 ± 0.97 pg/ml, *p* = 0.0295) (Figure 6). Significant reductions were still observed when considering only UC patients or only ADA treated patients (*p* = 0.0476 and *p* = 0.0103, resp.) (Figure 4 and Supplementary Figure 2). No changes were detected in EPO, neither in ERFE levels in our patients (Figure 6). Therefore, while downregulating IL-6 production, in our cohort, anti-TNF did not appear to influence the EPO-ERFE axis.

**Figure 7:**
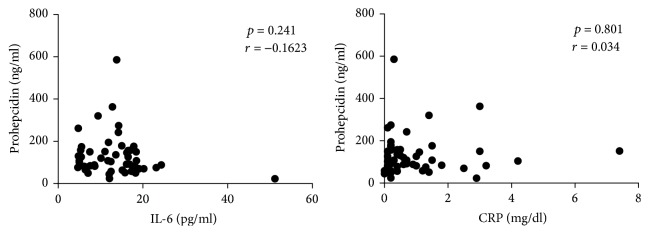
Correlation between IL-6 and CRP versus prohepcidin in CD patients. Statistical analysis was performed using Pearson's correlation coefficient.

**Figure 8:**
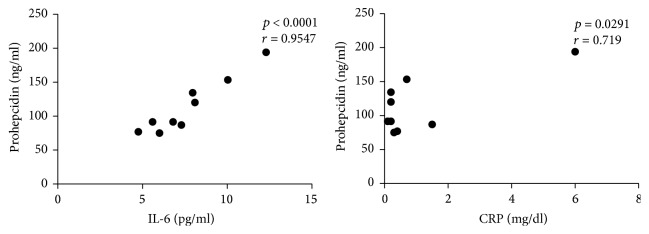
Correlation between IL-6 and CRP versus prohepcidin in UC patients. Statistical analysis was performed using Pearson's correlation coefficient.

Then, we tried to analyze the potential correlation between the circulating levels of IL-6, prohepcidin, and CRP. No significant correlation was detected when pooling together all patients or CD patients alone; however, when considering UC patients with a response to anti-TNF-therapy a statistically significant correlation between IL-6 and prohepcidin and between CRP and prohepcidin have been observed (*p* < 0.0001, *r* = 0.95 and *p* = 0.029, *r* = 0.72, resp.) (Figures 7 and 8). This may suggest a greater relevance of the IL-6-prohepcidin axis in UC or more simply a greater homogeneity of patients and disease features in UC, compared to CD.

## 4. Discussion

Anaemia is among the most frequent extraintestinal manifestations of IBD occurring in 6% to 74% of patients [1] and it has been suggested that anaemia is probably too common to be specifically recognized as a complication of IBD but it should be often seen as a consistent clinical feature of it [13].

The impact of anaemia on the quality of life in general patients and particularly in patients affected by IBD is profound [14, 15]. In particular, anaemia can debilitate IBD patients as much as abdominal pain or diarrhea and it may impair quality of life even in the absence of anaemia-specific symptoms [16]. Remarkably, the quality of life in IBD patients with anaemia may be as low as in anaemic patients with advanced cancer [16].

The mechanisms by which anaemia may develop are multiple, encompassing iron deficiency, chronic anaemia of inflammation, and vitamin deficiencies; in general, the system involving hepcidin and ferroportin expression is thought to play a major role in iron homeostasis and subsequent anaemia. Some studies have already been performed focusing on hepcidin levels in IBD. However, the data resulting from these studies have been very heterogeneous, with some indicating high hepcidin levels in CD and UC compared to controls [17], other showing no significant differences [18], still other reporting low levels in IBD, regardless of iron deficiency anaemia [19, 20]. These data may be explained considering the great heterogeneity among IBD patients included in these studies in terms of disease activity, clinical features, and iron status; in fact, it is worthy of note that whereas inflammatory states increase the production of hepcidin, its levels are drastically reduced during iron deficiency conditions [21]. Most recent data show that, in IBD patients, hepcidin appears to be a reliable marker for ACD [22], while not being an optimal indicator of IBD clinical activity [23]. As a matter of fact, the concomitant existence of intestinal inflammation and iron deficiency may lead to a great variability in hepcidin levels in IBD patients.

Therefore, given the prevalence of anaemia and its impact on the quality of life in IBD, we studied prohepcidin levels and iron homeostasis and their potential modifications induced by anti-TNF treatment in a group of patients with IBD. Interestingly, since we compared prohepcidin circulating levels from single patients at different timepoints, our methodological approach allowed us to overcome the great interpatients variability of the circulating levels of this protein.

In our series, when all patients with Crohn's disease and ulcerative colitis treated with anti-TNF were analyzed, serum prohepcidin was significantly decreased before W6 administration compared with baseline levels at *T*_0_. Overall, the decrease in serum prohepcidin levels paralleled the decrease in the clinical activity indexes, indicating control of inflammation. These observations were supported when patients were divided into the two diagnoses although the small number of UC patients enrolled and the more heterogeneous clinical responses were probably responsible for not reaching a statistical significance. Indeed, the data obtained on UC population may be useful to determine the sample size for future studies, focused on UC.

Consistently with the decrease in clinical activity indexes, we observed a significant reduction of CRP and ferritin, two acute phase proteins. These data suggest a strong correlation between abnormal iron homeostasis, inflammation, and disease activity in our patients with inflammatory bowel disease.

As anti-TNF therapy reduced circulating prohepcidin and ferritin, we detected a substantial improvement of iron homeostasis, associated with an increase in haemoglobin production.

This observation is in agreement with previous reports performed in IBD and other chronic inflammatory conditions, where anti-TNF therapy ameliorated patients' anaemia; as an instance, it has been shown that anaemic patients affected by CD increase their haemoglobin levels, when obtaining a clinical response to Infliximab administration [24]. In addition, Davis et al. demonstrated a dose-dependent improvement of anaemia in a cohort of rheumatoid arthritis patients undergoing Infliximab infusions [25]. More recently, Bes et al. performed a similar study in ankylosing spondylitis, again showing a significant improvement of haemoglobin levels in patients treated with both Infliximab and Adalimumab, compared to those treated with nonbiological agents [26]. Overall, authors predominantly ascribed this beneficial effect to the abrogation of TNF-mediated suppression of bone marrow erythropoiesis; however, in these studies, the possible interplay between TNF modulation and hepcidin production and its role in the regulation of anaemia of chronic disease were not taken into account.

As a matter of fact, since it has been largely demonstrated that hepcidin acts as an acute phase reactant, it is not surprising that its levels may be deeply influenced by potent anti-inflammatory agents such as anti-TNF drugs. Indeed, we believe that our data strongly suggest that the anti-inflammatory action of anti-TNF is responsible for the beneficial effect on haemoglobin levels through the inhibition of hepcidin production. In fact, hepcidin production is increased up to 100-fold in anaemia of inflammation. Moreover, it has been recently demonstrated on an animal model of colitis that the induction of intestinal inflammation promotes hepcidin production, in a microbiota dependent-fashion [27]. TNF is a pivotal cytokine involved in the pathogenesis of anaemia of chronic disease, by inhibiting proliferation and differentiation of erythroid progenitor cells and by inducing apoptosis of red cells precursors; however, a wealth of data clearly demonstrates that TNF is a potent inhibitor of hepatocyte production of hepcidin [2, 28]. Some indirect pathway might be hypothesized in order to explain our data. Therefore, we tried to obtain some insights on which hepcidin regulation pathway could be involved in mediating anti-TNF effects in our series. Thus, we measured the circulating levels of IL-6, erythropoietin, and erythroferrone. In fact, IL-6 was shown to be a prominent inducer of hepcidin. In healthy volunteers infused with IL-6, urinary excretion of hepcidin was increased several folds within two hours after infusion [28]. It has also been shown that TNF potently induces IL-6 production by macrophages, potentiating inflammatory responses. Conversely, several papers demonstrate that anti-TNF monoclonal antibody administration potently and promptly reduces circulating levels of IL-6 and other proinflammatory cytokines [29–31]. Therefore it might be hypothesized that the modulation of prohepcidin levels we observed following anti-TNF administration is obtained throughout an indirect pathway, likely involving anti-TNF-mediated downregulation of known hepcidin-inducing cytokines, such as IL-6. Supporting this hypothesis, Emery et al. demonstrated that Golimumab, another anti-TNF monoclonal antibody, promoted a significant reduction of urinary hepcidin in rheumatoid arthritis patients. This effect correlated with a consensual decrease of CRP and, remarkably, of IL-6 [32]. Consistently, we detected in our patients a significant reduction of IL-6 levels, suggesting that the TNF-IL-6 axis may be relevant in modulating hepcidin production during anti-TNF therapy. Nonetheless, our data are primarily descriptive in nature; therefore, additional “in vitro” and “ex vivo” observations, aimed at mechanistically dissecting the TNF-IL-6-hepcidin axis, are needed to confirm the importance of this pathway during anti-TNF therapy.

ERFE is a newly identified hormone, produced by erythroblasts, following EPO stimulus, which blocks hepcidin production [6, 33, 34]. ERFE has a role in the recovery from anaemia of inflammation, as demonstrated in a mouse model of ACD induced by injection of heat-killed* Brucella abortus*; in this model, through the suppression of hepcidin, ERFE contributes to the recovery from ACD [33].

EPO is a growth factor essential for erythroid progenitors and precursors and is produced in the kidney in response to hypoxia. Interestingly, Bergamaschi et al. demonstrated an increase of EPO, after Infliximab treatment in IBD patients [24]; thus, we anticipated anti-TNF blockade to restore EPO production and induce ERFE, further reducing hepcidin levels. Unexpectedly, the measurement of EPO and ERFE in our cohort did not show significant changes after Infliximab and Adalimumab therapies. This may be due to the fact that our patients, overall, showed mild anaemia; thus, they may not have been exposed to a hypoxic stimulus, substantial enough to significantly increase EPO levels. Indeed, further studies, on a broader population, are warranted to evaluate the relevance of the erythropoietin-erythroferrone axis in IBD patients, not only in the context of anti-TNF therapy.

Taken together, our data show that anti-TNF therapy significantly improves iron metabolism and, subsequently, anaemia in IBD. This effect appears to be primarily related to the modulation of the cytokine network, leading to a relevant decrease of hepcidin, a master regulator of anaemia of chronic disease. Therefore, in IBD patients anti-TNF agents seem to play a role in ameliorating anaemia of chronic disease during a 6-week treatment; on the other hand, in a long-term treatment, anti-TNF therapy may also improve iron deficiency anaemia, throughout the induction of mucosal healing. However, there is still the possibility that clinical improvement among IBD patients is not necessarily linked to the restoration of iron homeostasis.

## Supplementary Material

Prohepcidin and Iron Status Markers, Erythroferrone (ERFE), Erythropoietin (EPO), and Interleukin- (IL-) 6 concentrations were measured as described in Matherial and Methods in the main text. The data obtained were analyzed according to the Anti-TNF therapy i.e. Infliximab or Adalimumab.

## Figures and Tables

**Table 1:** Demographic and clinical characteristic of enrolled patients.

Diagnosis	CD	UC
Number of patients	16	5
Age (mean ± SD)	35.2 ± 13.9	55.6 ± 7.1
Sex (male/female)	5/11	2/3
Location CD		
Ileal	1	—
Colonic	4	—
Ileocolonic	11	—
Upper GI involvement	0	—
Extent UC		
Proctitis	—	0
Left sided colitis	—	1
Extensive colitis	—	4
Disease behavior CD		
Nonstricturing, nonpenetrating (B1)	14	—
Stricturing (B2)	0	—
Penetrating (B3)	0	—
Perianal disease	2	
Previous resective surgery	2	—
Concurrent treatment		
Mesalazine (# of pts)	1	4
Steroids (# of pts)	6	2
Immunosuppressors (# of pts)	7	1

**Table 2:** Patients' clinical scores.

CD patients					
Pts #		*T*_0_ HBI	W2 HBI	W4 HBI	W6 HBI
*IFX treatment*					
1		11	5	—	2
2		8	3	—	1
3		8	2	—	1
4		7	4	—	3
5		6	3	—	0
6		5	2	—	2
7		8	3	—	3
8		3	2	—	1
9		2	0	—	0

*ADA treatment*					
10		10	8	8	5
11		10	5	7	5
12		3	1	0	0
13		7	2	2	2
14		6	4	4	3
15		9	1	0	0
16		5	4	3	3

UC patients					
Pts #	*T*_0_ MS	*T*_0_ PMS	W2 PMS	W4 PMS	W6 PMS

*IFX treatment*					
17	7	5	0	—	0
18	7	5	7	—	8
19	9	7	2	—	7
20	9	7	0	—	0
21	7	4	0	—	0

HBI, Harvey-Bradshaw Index; MS, Mayo score; PMS, partial Mayo score.

## Nonstandard Abbreviations

ACD: Anaemia of chronic disease
ERFE: Erythroferrone
EPO: Erythropoietin
DMT-1: Divalent metal transporter-1.
