# Supplementary figures and images for: Anti-TNF-Mediated Modulation of Prohepcidin Improves Iron Availability in Inflammatory Bowel Disease, in an IL-6-Mediated Fashion
Source: Can J Gastroenterol Hepatol. 2017 Jan 16;2017:6843976. doi: 10.1155/2017/6843976 (PMC5278212; doi:10.1155/2017/6843976)

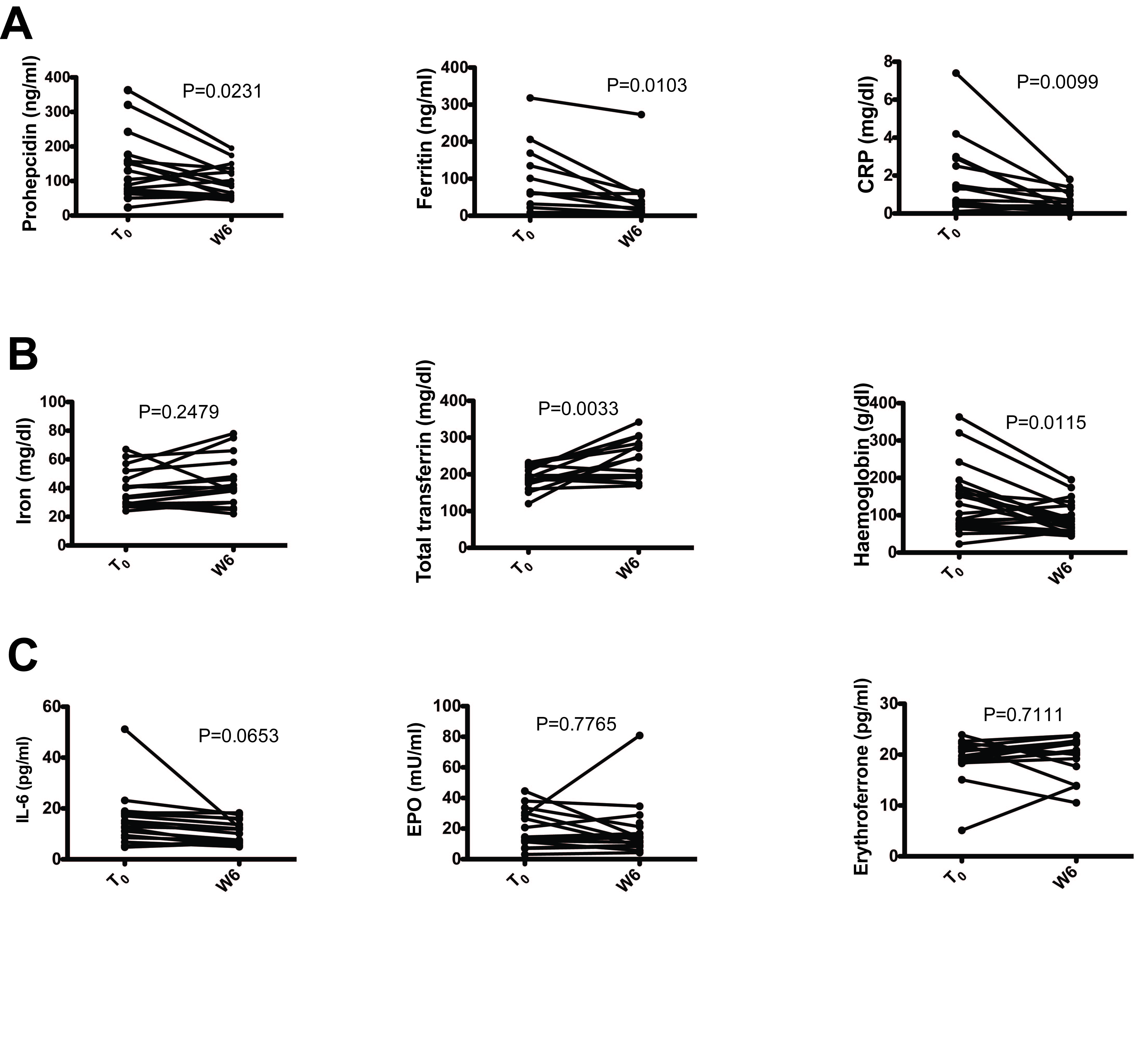

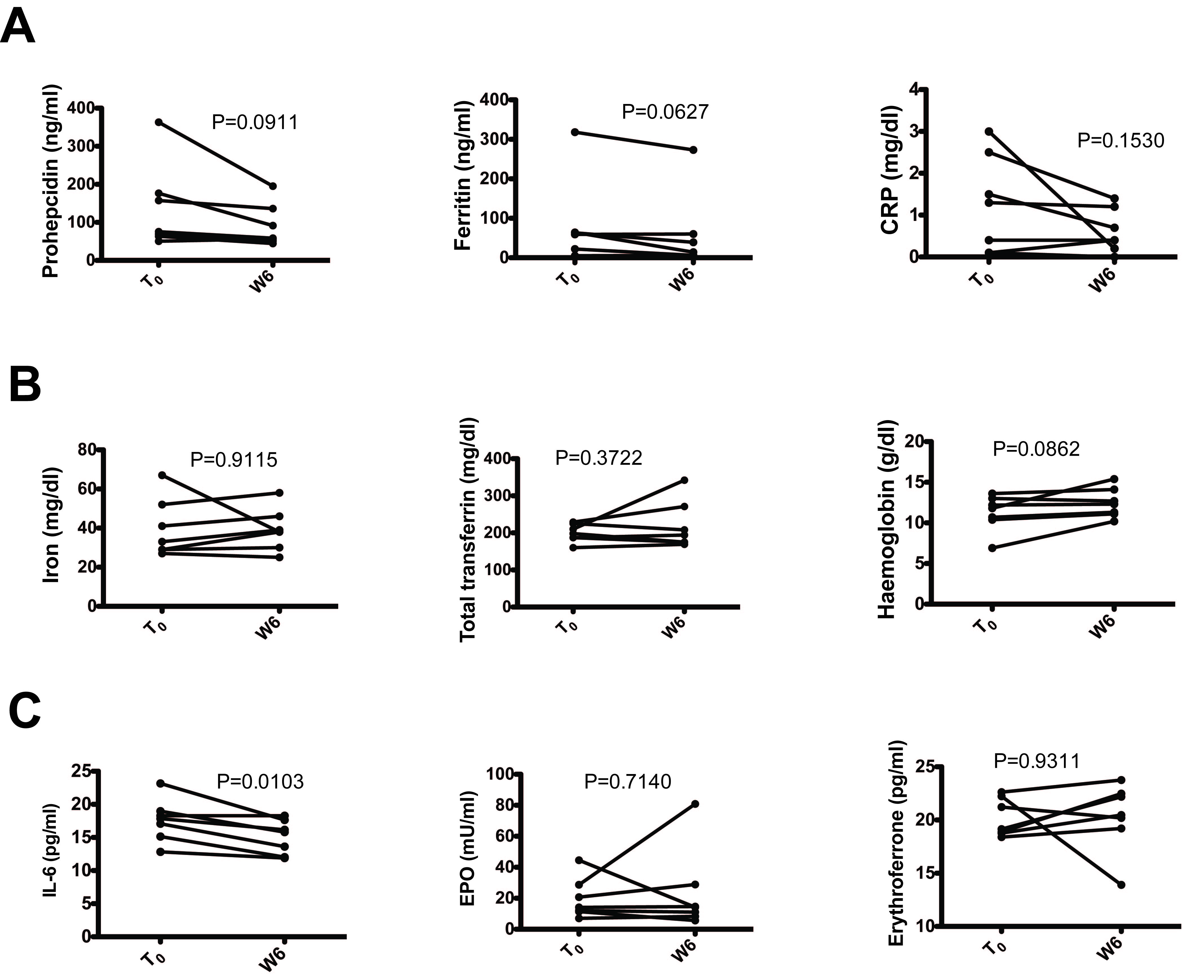

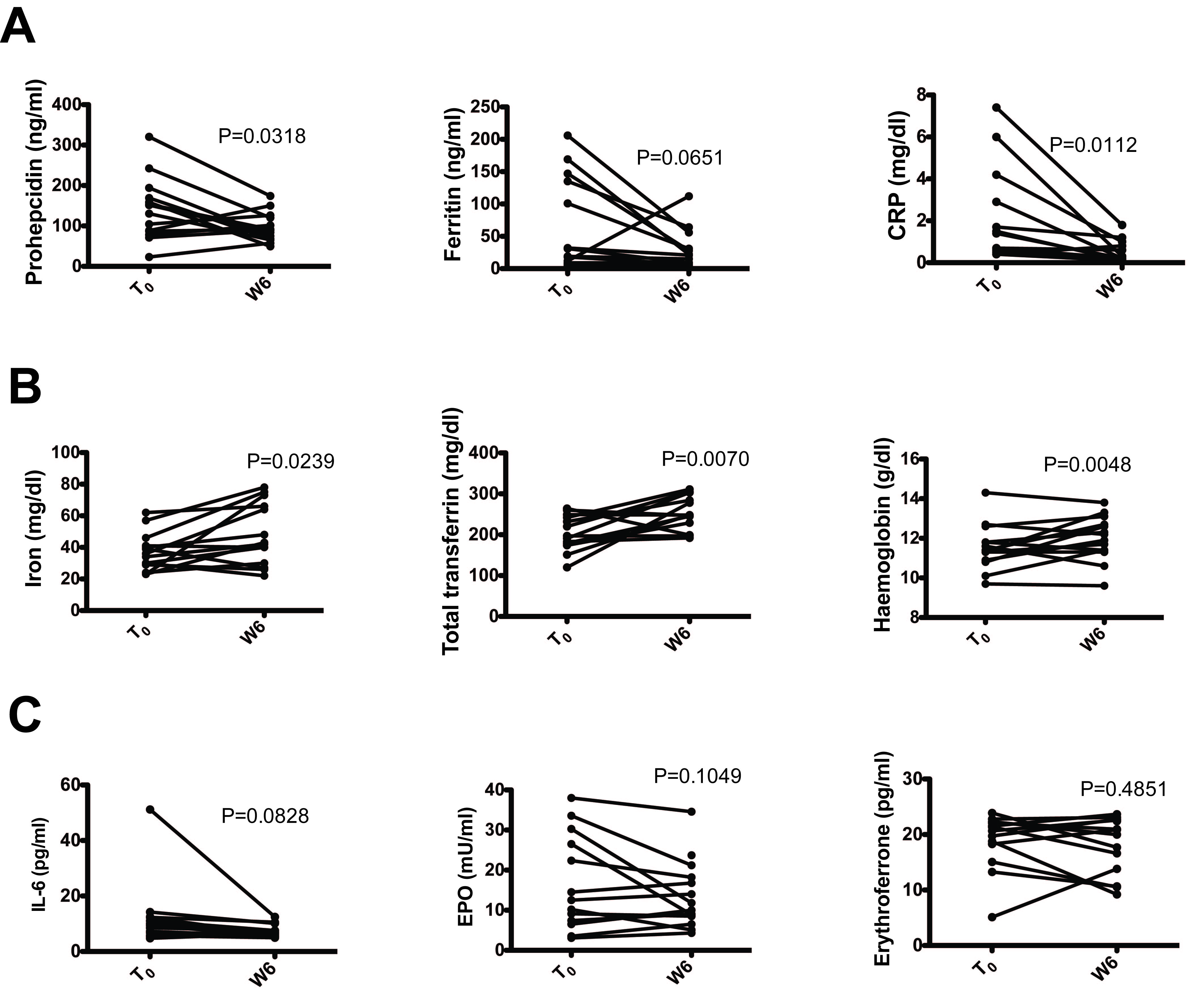

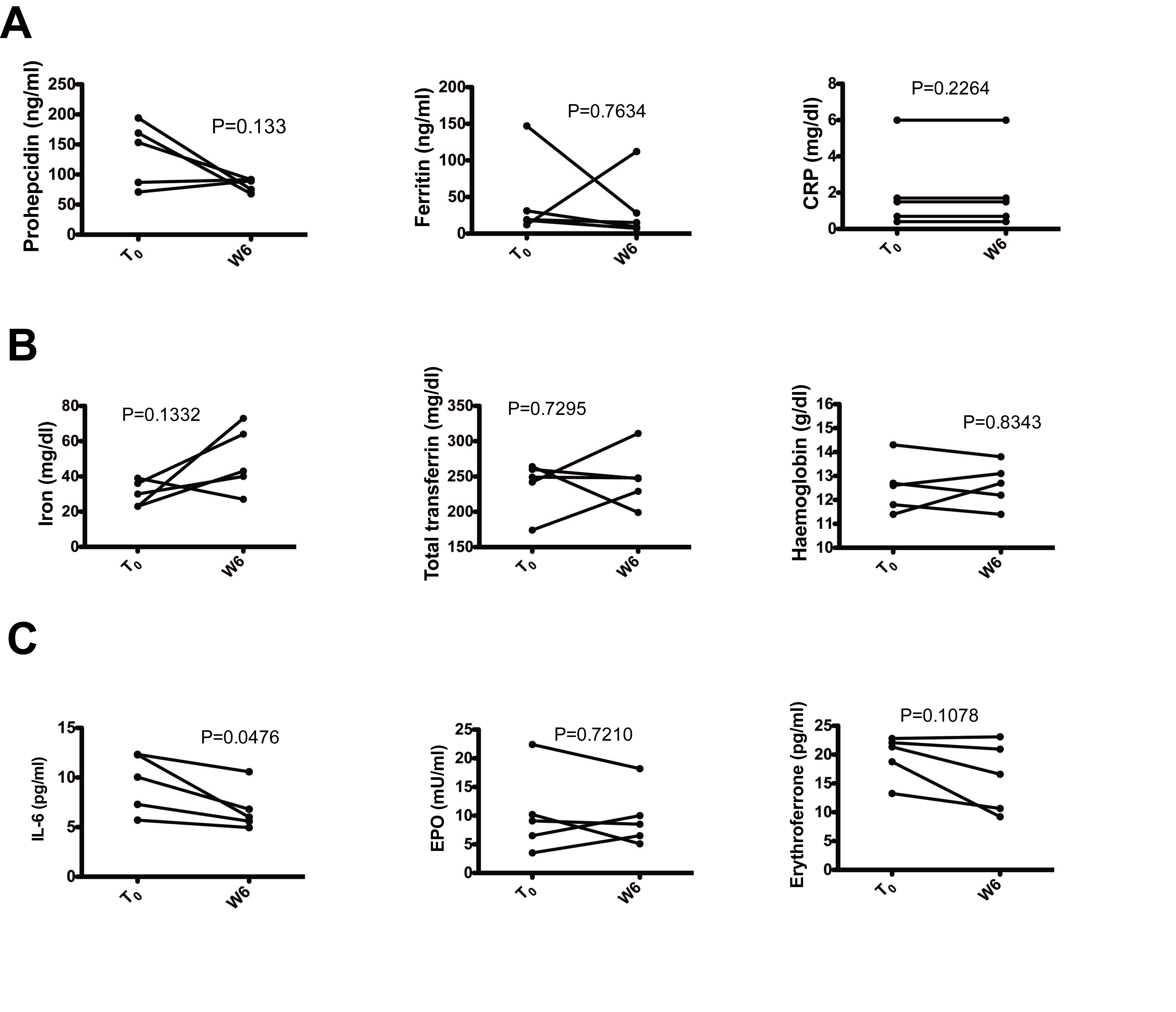

Supplement: Supplementary file 1 — Prohepcidin and Iron Status Markers, Erythroferrone (ERFE), Erythropoietin (EPO), and Interleukin- (IL-) 6 concentrations were measured as described in Matherial and Methods in the main text. The data obtained were analyzed according to the Anti-TNF therapy i.e. Infliximab or Adalimumab. [file 6843976.f1.docx]
